# Consensus-building during the Becoming Breastfeeding Friendly (BBF) initiative in Samoa: A qualitative content analysis

**DOI:** 10.1371/journal.pgph.0001725

**Published:** 2023-04-24

**Authors:** Katherine Daiy, Kendall Arslanian, Courtney Choy, Analosa Manuele-Magele, Christina Soti-Ulberg, Amber Hromi-Fiedler, Nicola Hawley, Rafael Pérez-Escamilla

**Affiliations:** 1 Department of Anthropology, Yale University, New Haven, Connecticut, United States of America; 2 Department of Social and Behavioral Sciences, School of Public Health, New Haven, Connecticut, United States of America; 3 Department of Chronic Disease Epidemiology, Yale University, New Haven, Connecticut, United States of America; 4 Ministry of Health, Apia, Samoa; African Population and Health Research Center, KENYA

## Abstract

The Becoming Breastfeeding Friendly (BBF) initiative provides countries with an evidence-based toolbox to improve the national breastfeeding environment by assessing and developing a plan to effectively scale up well-coordinated national policies and programs. BBF is carried out by a multi-institutional, intersectoral committee of experts, convening across five committee meetings designed to produce policy recommendations that can be implemented in the country’s context. Samoa successfully completed the BBF initiative in 2018, resulting in the institution of breastfeeding policy in hospitals and the establishment of lactation rooms within government ministries. An important step in informing the success of future breastfeeding scale-up initiatives is understanding how consensus is built. This study aimed to investigate how the BBF Samoa committee built consensus. We conducted a content analysis of audio recordings of three BBF Samoa meetings (meetings 1, 2 and 4), meeting minutes, and meeting notes using an *a priori* operational consensus-building framework. We used a combination of deductive and inductive approaches to: a) evaluate the data against existing, *a priori* criteria for consensus-building and b) identify emergent ways in which the BBF Samoa committee may have achieved consensus. We identified 6 themes, 2 meta-subthemes, and 16 subthemes. The 6 themes, largely defined by the *a priori* framework, represented key components of successful consensus-building. The 2 meta-subthemes described two overarching methods of consensus-building: “process-led” (i.e., inherent to the BBF process itself) and “organic” (unique/specific to the committee). Lastly, the 16 subthemes described more specific ways that the committee reached consensus. The detailed manualization of the BBF process, its reliance on data, and its transparent and engaged committee process were key for reaching consensus on BBF scores and recommendations in Samoa. Our study contributes to the understanding of how effective breastfeeding policy recommendations are made, using a methodology that can be applied beyond the topic of breastfeeding.

## Introduction

Serving as “personalized nutrition” for the developing infant, breastfeeding is a powerful intervention for improving child survival, health, and development. However, breastfeeding prevalence remains low; in low and lower-middle-income countries, 50–52% of infants under 6 months of age are exclusively breastfed [1]. The Becoming Breastfeeding Friendly (BBF) initiative provides countries with an evidence-based toolbox to improve the friendliness of the national breastfeeding environment by identifying policies and programs that can be effectively scaled up and coordinated, taking the national context into account.

The BBF initiative is grounded on the Breastfeeding Gear Model, which proposes 8 essential elements or gears for the national breastfeeding “engine” to function well: 1) advocacy, 2) political will, 3) legislation and policy, 4) funding and resources, 5) training and program delivery, 6) promotion, 7) research and evaluation, and 8) coordination, goals and monitoring [2]. Each of the eight gears are expected to work in harmony to increase the friendliness of a nation’s breastfeeding environment. A country’s implementation of the BBF initiative includes at least five meetings held by an intersectoral committee of experts from government ministries, civil society, international agencies, and academic institutions. The BBF committee was comprised of 20 members who represented diverse organizations including the Ministry of Women, Community and Social Development (MWCSD); the Ministry of Education, Sports and Culture (MESC); the Ministry of Commerce, Industry and Labor (MCIL); the Public Service Commission (PSC); Samoa Red Cross; the National University of Samoa; and the National Health Service (NHS). These stakeholders are expected to work together across each of the five meetings to assess the readiness (for scale-up) of a country’s breastfeeding environment and to develop recommendations for improving breastfeeding policies and programs [3, 4].

A key requirement for the BBF process to be successfully implemented is for the committee to reach consensus every step of the way on the BBF scores and recommendations. Consensus-building refers to a practice in which stakeholders, selected to represent different interests, join for face-to-face dialogue to address a policy issue of common concern [5]. Consensus is defined as the process by which a group reaches agreement about the best solution to a problem or the best choice among alternative options [6]. There are multiple process criteria for effective consensus-building defined by Innes and Booher [5]. First, consensus-building should include representatives from many different interests and perspectives (“Diverse perspectives are incorporated,” Table 1). It must also be adaptive, evolving and self-organizing, and may spur smaller discussion groups (“Adaptive and self-organizing discussion,” Table 1). Another criterion for effective consensus-building is engagement. Stakeholders must be involved in in-depth discussions and active learning (“Stakeholders are actively engaged,” Table 1). Moreover, impactful consensus-building processes must allow for challenges to the status quo and foster creative approaches to presented problems (“Challenges to status quo, creative solutions,” Table 1). Finally, effective consensus-building incorporates high-quality information of many varieties while ensuring a unified interpretation of this information from different stakeholders and seeks consensus only after extensive discussion and exploration of the issues at hand high-quality ("Unified interpretation of high quality data"; "Seeks consensus only after extensive discussion"; Table 1). We used these different components of consensus-building as a guiding framework (i.e., what we call *a priori* themes) for deductive analysis of the BBF Samoa meetings.

**Table 1:** *A priori* themes using Innes and Booher’s [5] process criteria for effective consensus-building.

Diverse perspectives are incorporated
Adaptive and self-organizing discussion
Stakeholders are actively engaged
Challenges to status quo, creative solutions
Unified interpretation of high-quality data
Seeks consensus only after extensive discussion

**Fig 1:**
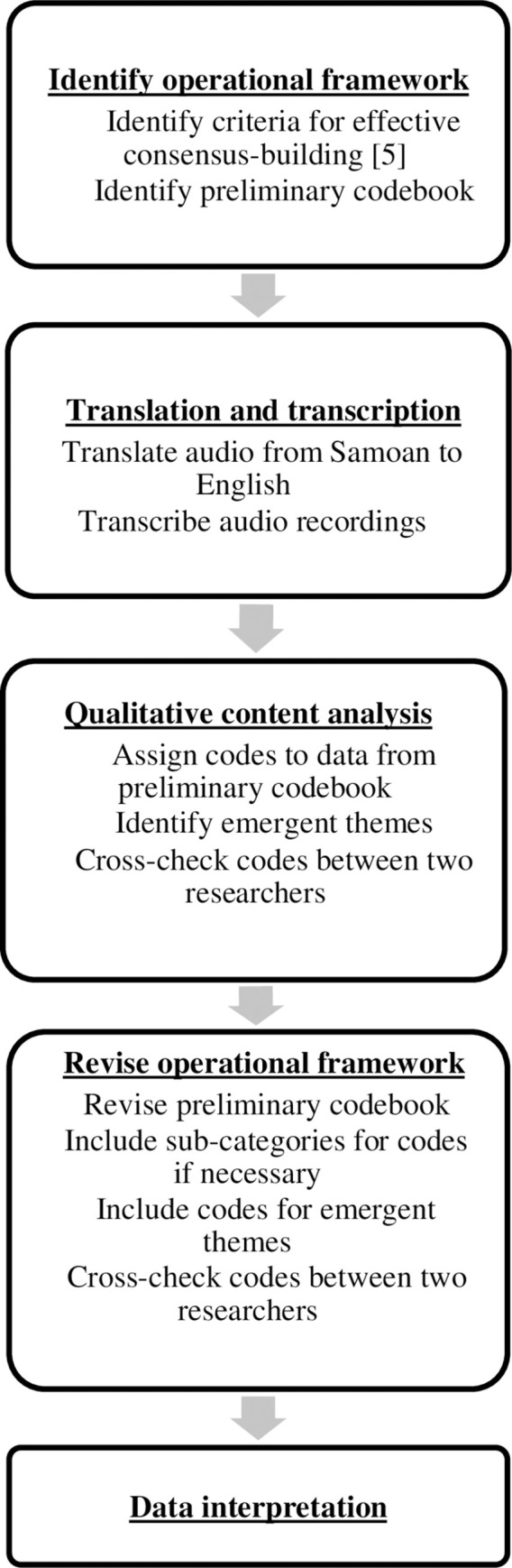
Study design.

By design, the BBF process encourages consensus-building processes by convening stakeholders who hold different perspectives to assess the state of their national breastfeeding environment and decide on recommendations. Between January and August 2018, Samoa implemented BBF with the help of a 20-member committee of breastfeeding experts, including academics, policymakers, and public health officials across governmental ministries, universities and other institutions [7]. Samoa is an optimal case study for understanding consensus-building within BBF due to its successful implementation. The objective of this study is to describe and characterize the consensus-building process employed by committee members throughout the 5-meeting process of the 2018 Becoming Breastfeeding Friendly (BBF) initiative in Samoa. We conducted a content analysis of audio recordings of three meetings (meetings 1, 2, and 4) and associated meeting minutes. Understanding the consensus-building process in BBF Samoa will aid in characterizing the real-world implementation of the BBF initiative, as well as providing insights that can be used to optimize the BBF process, as it continues to be implemented worldwide. Our study design is described in Fig 1.

## Methods

### BBF process

The BBF process has been described in detail previously [3, 4]. Briefly, the BBF process involves a) *scores* that assess the national breastfeeding environment across different gears and b) policy *recommendations* to improve breastfeeding systems, services, and outcomes. Across the eight gears, there are 54 benchmarks that can be scored using data collected by the committee members to assess the country’s progress. Case studies, or examples of BBF implementation in other countries, are provided to BBF committee members to assist them in understanding how to score benchmarks and develop corresponding policy recommendations. All benchmark scores within each gear are summarized into a *gear score*, which represents the level of progress reached by a country on the goals surrounding a particular gear. A *total country score*, or BBF index score, is a representation of the friendliness of a country’s breastfeeding environment and is calculated by combining all gear scores [4]. An in-country BBF committee also discusses and develops *priority policy recommendations*, which are specific recommendations for decision-makers that are designed to improve breastfeeding systems, services, and outcomes nationwide.

### Setting and BBF Samoa committee

Samoa is a lower-middle income country in the West Pacific region and has a population of approximately 197,000 people. Samoa has a high burden of obesity and related non-communicable disease; as such, breastfeeding and its protective effects on non-communicable disease would be beneficial in this context [8]. The vast majority (93%) of women aged 25–64 have overweight or obesity [9] and maternal and child malnutrition are also present [10]. Breastfeeding rates are high in Samoa (94% of all infants are breastfed); only 70% of infants under 6 months are exclusively breastfeed, less than the 100% recommendation [11]. However, in Samoa, breastfeeding is viewed as protective and beneficial for mothers and infants [12]. Between January and August 2018, the Samoan Ministry of Health led the BBF initiative in order to scale-up nationwide breastfeeding policy and programs and improve the national breastfeeding friendly environment [7]. As indicated in the BBF operational manual, consultations with experts outside the committee were allowed as needed.

### Data analysis

We conducted content analysis of audio recordings from three of the Samoa BBF committee meetings (meetings 1, 2, 4) and meeting minutes from Meeting 3. Audio transcripts were the primary source of data and meeting minutes were used to complement the audio transcripts. The audio recordings from Meeting 3 could not be located so we exclusively used the meeting minutes to analyze content from that meeting. Because Meeting 5 was a day-long public event and consisted mostly of presentations of recommendations and scoring–the end results of the consensus-building process–we did not analyze the content of this meeting. Committee members used a combination of Samoan and English in their discussions and presentations. As such, audio recordings were transcribed verbatim in Samoan and then translated into English by two Samoan research assistants who were fluent in Samoan and English and familiar with the BBF process.

A codebook for content analysis was developed through a deductive approach, which mapped themes onto those in Table 1, derived from scholarly work on effective consensus-building [5], and an inductive approach, which allowed the data to determine the emergent themes. NVivo software [13] was used to code all audio transcripts and meeting minutes. NVivo was also used to count all words when committee members discussed each BBF gear as a proxy for how much time committee members spent discussing the gear. The coding process involved a first meeting to discuss the initial codebook in Table 1 (using Innes and Booher’s [5] framework) and to ensure that both coders understood the meanings of the different codes. Following, two authors (KD and KA) independently coded the first BBF meeting transcript and met to discuss any discrepancies in the deductive coding and as they adhered to the *a priori* themes from Table 1. The two authors also discussed any emergent themes they found using an inductive method, which were added to the codebook. They used the initial codebook, adding and refining it with the same deductive and inductive process for the meeting 2 and 4 transcripts. The codebook was then discussed with the other authors to reach additional consensus. Finally, the two authors re-evaluated all transcripts with the finalized codebook and met together to compare codes, resolve coding differences, and finalize coding decisions. Altogether, final themes from deductive coding in Table 1 were identified and emergent themes were identified that were unique to consensus-building during the Samoa BBF process.

### Ethics statement

The study protocol for this secondary data analysis was approved by the Samoa Ministry of Health’s Health Research Committee Institutional Review Board (September 2020). This secondary data analysis was exempt from human subjects’ research review by Yale University’s Institutional Review Board because the project queried participants only for their expert opinions as public figures or program managers, which does not constitute the definition of human subjects’ research at Yale. All participants agreed for their names to be acknowledged in this manuscript and the Samoan Government HIC approved of this submission. The audio recordings were taken for the initial BBF project and did not require formal consent given that the project did not constitute human subjects research according to the Institutional Review Boards at Yale and at the Samoa Ministry of Health [7].

## Results

**Fig 2:**
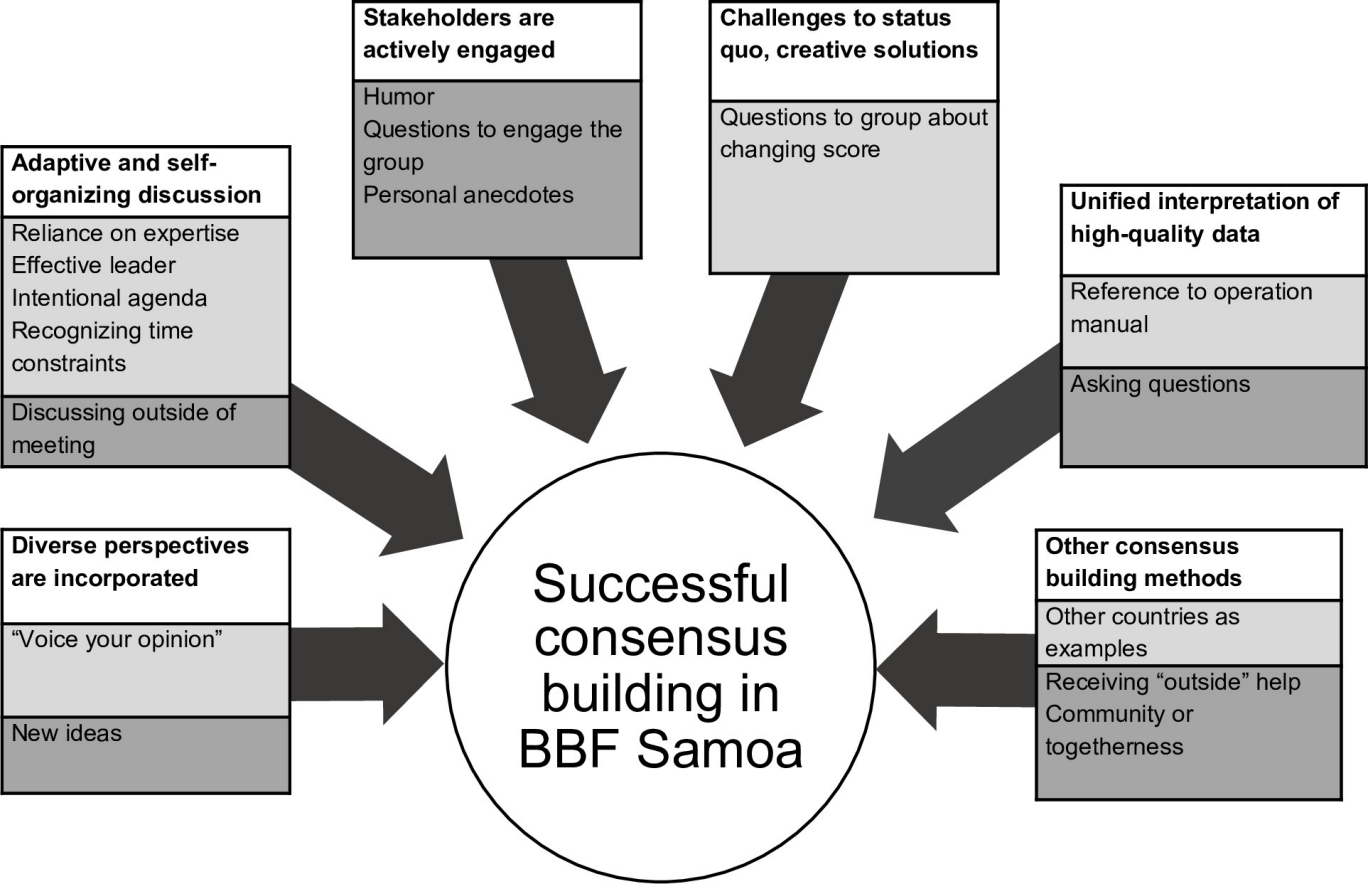
Organization of themes and subthemes. Subthemes are organized into “process-led” (light gray boxes) and “organic” (dark gray boxes) aspects of consensus-building.

We identified 6 themes, 2 meta-subthemes, and 16 subthemes. The 6 themes, were defined by Innes & Booher’s [5] framework for successful consensus-building (Table 2; Fig 2). We found evidence for all subcomponents of their framework, except “Seeking consensus only after extensive discussion,” which we did not identify in the meeting transcripts. Under the 6 themes, we identified 2 meta-subthemes–“process-led” and “organic” consensus-building. We define these, respectively, as 1) when the committee utilized the designated BBF toolkit or followed the BBF process to build consensus, which included relying on committee members expertise or by following intentional agendas (“process-led”), and as 2) when the committee utilized other consensus-building tactics, such as by using humor or broaching feelings of community and togetherness (“organic”). Lastly, we identified 16 subthemes under the themes and meta-themes, which are reported in depth below.

**Table 2:** Thematic organization.

*Theme*	*Meta-subtheme*	*Subtheme(s)*
Diverse perspectives are incorporated	Process-led	“Voice your opinion”
	Organic	New Ideas
Adaptive and self-organizing discussion	Process-led	Committee members’ expertise Intervention of leader Intentional agenda Recognizing time constraints
	Organic	Discuss outside of meeting
Stakeholders are actively engaged	Process-led	*None*
	Organic	Humor and sarcasm Questions to engage the group Personal anecdotes
Challenges to status quo, creative solutions	Process-led	Questions to the group about changing the score
	Organic	*None*
Unified interpretation of high quality data	Process-led	Reference to operational manual
	Organic	Asking questions
Other consensus-building methods	Process-led	Other countries’ as examples
	Organic	Receiving “outside” help Community or togetherness

**Fig 3:**
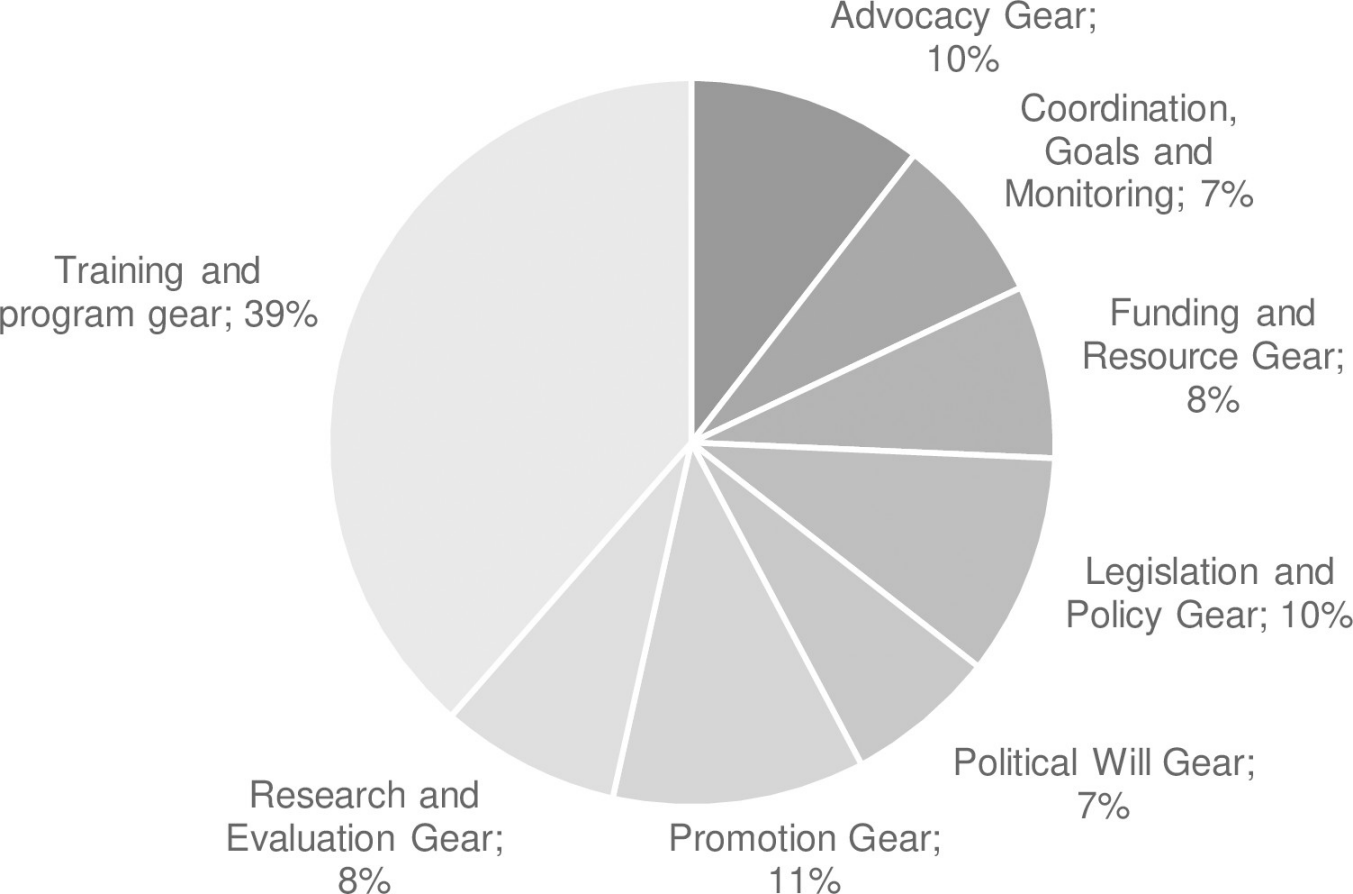
Percent of words discussing each BBF Gear from all meeting transcripts as a proxy of meeting time spent discussing each gear.

We additionally found that the Advocacy and Training and Program Delivery Gears were discussed most often by committee members and, as a result, were coded the most frequently among the available transcripts (Fig 3). We provide this information as a point of comparison for past and future BBF meetings; it may be that some gears take longer to discuss or that time spent on certain gears is related to better BBF outcomes and consensus-building.

### Diverse perspectives incorporated

The incorporation of diverse perspectives was consistently present throughout the BBF process, and open-ended discussion was a key component of the meeting process. In fact, a large proportion of time in the analyzed transcripts (just under half; 45%) reflected open-ended discussion, both spontaneous and scheduled, among the committee members. This component was reflected in the meetings through the frequent sharing of new ideas (“New Ideas”), as well as the tendency of speakers to ask for everyone to “voice their opinions” (“Voice your opinion”). The theme of “New Ideas” reflected an organic, committee-specific aspect of consensus-building that went beyond the rules and structure of the BBF process, while “Voice your opinion” reflected a more purposive aspect of consensus-building that was already prescribed by the BBF process.

### Process-led aspects of consensus-building

#### “Voice your opinion”

Throughout the meetings, the BBF leader or another committee member would open the room for discussion, explicitly requesting that everyone share their true opinions on certain topics. These calls for opinions and ideas were embedded into the BBF process, such that presenters and committee leaders were suggested to frequently gauge committee-wide opinions on topics. An example from Meeting 1 (discussion on the Advocacy Gear) is shown below:

*“Voice out your opinion if that score is suitable or change it*? *And what are the reasons we want to change it?”*

### Organic aspects of consensus-building

#### New ideas

This subtheme referred to how members presented new ideas to the group. Committee members frequently interjected in presentations and open-ended discussions with new ideas, often regarding the development of recommendations and the overall scale-up of breastfeeding in Samoa. These new ideas emerged in all parts of the BBF process, even during times where open-ended discussion was not on the intentional agenda (e.g., during PowerPoint presentations). Thus, we categorize this as an “organic” aspect of consensus building. As an example of a new idea, in Meeting 2, when the committee discussed the scoring for the final benchmark of the Promotion gear (“The government has raised awareness about breastfeeding”), a committee member suggested following up with workplaces about their efforts to implement breastfeeding promotion programs:

*Committee member*: *“Is there a follow-up plan for the organizations to know what you are trying to deliver*? *Like within particular workplaces [where breastfeeding is promoted], so that you know what you are trying to do [promote breastfeeding] is effective?”*

#### Adaptive and self-organizing discussion

Another key component of successful consensus-building was self-organizing discussions, such that participants in the process decided on objectives, tasks and other items together [14]. We found that the BBF process adhered to this key style through the a) reliance on committee member’s expertise to answer questions and address points of disagreement throughout the meetings (“Reliance on expertise”); b) the effectiveness of the leader (BBF committee chair) when there were uncertainties in the discussion (“Effective leader”); c) intentional agendas; d) recognition of time constraints; and e) the discussion of items outside of the meeting. The subthemes “Reliance on expertise”, “Effective leader, “Intentional agendas,” and “Time constraints” all reflected the purposive aspects meta-theme of consensus-building, while “Discussion outside meetings” reflected organic, committee-specific meta-theme of the consensus-building process.

### Process-led aspects of consensus-building

#### Reliance on expertise

The expertise of the BBF committee members on the topics of breastfeeding science, policy, advocacy, among other things, was relied upon to make decisions during the BBF process. Both the BBF committee leader and committee members held extensive knowledge in areas relevant to the various gears and often would interject or add to open-ended discussions to help the committee arise at decisions.

*Committee member 1*: *Then comes our benchmark number nine. Number nine talks about the coordination and the integration of breastfeeding training programs. We have a 20-hour breastfeeding counselling training, which collaborates with the Ministry of Health and the National University of Samoa for staff members working in the maternity services. [However], we put [this training] as a need and [we must] find out from [name redacted] how often and how many times we have offered this training since 2006*.

*Committee member 2*: *This training started in 2006, then was held again in 2014 and 2016*.

*Committee member 3*: *Just to add, I think there was a change in training in 2009, 2011, 2014, and last year*.

#### Effective leader

During the meetings, the BBF committee chair (CSU) oftentimes intervened during presentations or during open-ended discussions to correct assumptions and improve the committee’s understanding of certain topics. The extensive knowledge of the leader regarding breastfeeding in Samoa allowed many questions to be answered and ensured that all committee members had full understanding of the various gears, benchmarks, and rules of the BBF process. This also ensured that decision-making in meetings was carried out smoothly and efficiently. The BBF process was set up such that a committee leader guided most of the process; thus, we designated this subtheme as the “purposive” meta-theme of consensus-building. One example shows how the committee chair gave feedback after a gear team’s presentation and asked them to consider other sources of data by the time of the next meeting:

*[Committee member finishes a presentation]*

Committee chair: *Thank you*. *Well done*. *Thank you for your efforts to be clear [about] the differences of the gear*. *I think almost you already know your scoring because throughout the presentation you’ve been saying that it must be major*. *I think the final thing for your team is to put what you really said in the next meeting*. *So the other thing you mentioned is that you do this and that*, *it all needs the source of data*, *sources of evidence*, *then bring it forward on the next event [next meeting]*. *But other than that*, *well done*.

#### Intentional agenda

The committee prepared an agenda for each BBF meeting, which was explained at the beginning and brought up when conversations diverged from the schedule. For example, when discussions were too lengthy, the BBF committee leader mentioned the original meeting agenda to keep the committee members on track. An example of how intentional agendas were established early in meetings is shown below from the introduction of Meeting 4 when the committee chair welcomed everyone and briefly outlined the agenda for the day.

*Committee chair*: *“The first one [agenda item] is to go back to our different Gears and reach consensus on the outstanding benchmarks [–] The following section will be a presentation, which will be conducted by [name redacted] regarding our surveys that we filled in over the last two weeks*. *It will give us a snapshot of what we all feel and what we all understand, and what actual recommendations we will prioritize and rank by the end of today*.

#### Recognizing time constraints

This subtheme illustrates how committee members recognized and dealt with time constraints during meetings, so that a strict schedule was adhered to and time was not wasted. Stating that presentations and discussions had to be done in a timely manner allowed for meeting efficiency and ensured that the decision-making process went smoothly. Two examples are shown below:

*Committee chair*: *“Okay, we are not going to wait any longer, because it is now 10 o’clock. I am so sorry for some people’s lateness. I know they have lots of things to do, especially Heads of Ministries and different Organizations.”*

*Committee chair*: *“Let’s move on. There is no time.”*

### Organic aspects of consensus-building

#### Discussing outside of meeting

This subtheme refers to how committee members mentioned that additional discussion was needed outside of the meeting or at another meeting. Discussing topics outside of committee meeting goes beyond the requirements of the BBF process, thus this was categorized as the meta-theme of organic consensus-building. An example from Meeting 2 on the discussion of the Advocacy Gear is below:

*Committee member*: *“So if there’s any comments, then we will work through it. Because it’s still a preliminary score, we can always change the score in the second meeting as well, but we leave this here at ‘two.’”*

In summary, self-organizing and adaptive discussion in the 2018 BBF process in Samoa was supported through several process-led and emergent methods, including guidance from strong leadership, the use of an intentional agenda, the recognition of time constraints, and the designation of additional time outside of meetings for discussion.

#### Stakeholders are actively engaged

Keeping stakeholders actively engaged, interested and “at the table” is another key component of a successful consensus-building process [5]. The BBF committee employed several methods to keep participants engaged, including the use of a) humor and sarcasm, b) open-ended questions that had the purpose of engaging participants and c) personal anecdotes. All these themes were “organic” and unique to the BBF Samoa committee. The themes are described below.

### Organic aspects of consensus-building

#### Humor

BBF committee members often employed humor during presentations or open-ended discussions. Often met with laughs from the audience, jokes were used to engage others, make points, and to “lighten the mood” of the meetings. Two examples are below:

*“Welcome everyone*. *Here we are now onto the gear which has many benchmarks. We are the most blessed team of all. [laughter from the committee]”*

*“So we’re going to the first gear*. *Can you read our first gear? [a long silence ensues] Okay! Very enthusiastic! [laughter from the committee]”*

#### Questions to engage the group

Questions were posed to the group with the goal of generating interest, participation or engagement. These questions–often rhetorical questions–asked committee members to consider their own personal experiences, encouraging them to relate to the tasks posed by BBF in a personal way. These types of questions were often used by the BBF committee leader to help keep participants engaged throughout the BBF process.

*How many of you in the room have four or five kids*? *There are many*.

#### Personal anecdotes

BBF committee members brought up and utilized personal anecdotes to elaborate on points of discussion. These anecdotes ranged from personal experiences regarding breastfeeding and infant care, to experiences working with breastfeeding-related health initiatives. Two examples are shown below:

“*Is there any special training for mothers that have problems with their breasts*, *which is why they couldn’t breastfeed their kids*? *Because it looks like these are the mothers who trainings are for*. *For example*, *the breast cannot provide any breast milk*, *even using the pump or medicine from traditional healers*…*Another [reason to breastfeed] is to [protect] from breast cancer*. *I just want to know some ideas or ways that can encourage [breastfeeding]…*, *because I have 8 kids*. *None have ever had a chance to breastfeed*.”

“*Hello and good health*. *[…] I have six kids*, *and I breastfed all six*. *I breastfed even when they were going to school*. *So today is very important because you must look at [what is healthy for the kids]*. *My daughter is now 16*, *and she is still strong and smart [in part because of breastfeeding]*.”

In summary, committee members were engaged in the BBF process through humor and sarcasm, questions to engage the group, and personal anecdotes, all of which emerged organically and facilitated a positive atmosphere for decision-making.

#### Challenges to status quo and creative thinking

Other key components of successful consensus-building are challenges to status quo and creativity in finding solutions to problems. For instance, committee members challenged other members’ scoring of gears and benchmarks when they did not feel it was accurate (“Question to the group about changing scoring”). This reflected the meta-theme of process-led consensus-building embedded in the BBF process since committee members were expected to interject with differing opinions about scoring of BBF gears and recommendations.

### Process-led aspects of consensus-building

#### Question to the group about changing scoring

In many cases, either the BBF committee leader or member asked the rest of the group if the scoring of a gear or benchmark should be changed. The purpose of this was to gather feedback and “check” the scoring for alignment with the committee’s consensus. For example, during a discussion on the Legislation and Policy Gear in Meeting 4, a committee member offered a discussion to change a score:

“*I think I’ll leave it to the floor; I’m not going to decide*. *[…] I’ll leave it to the floor for any or further comments or just put [it] under Partial*. *But definitely*, *[our score] is not zero*.”

#### Unified interpretation of high-quality data

The BBF process also involved the incorporation of high-quality, diverse data and unified interpretation of the data. Committee members often referenced the operational manual when there were misunderstandings (“Reference to operation manual”), a “process-led” method of consensus-building, and asked questions for clarification (“Asking questions”), an “organic” method of consensus-building. These methods allowed everyone on the committee to be “on the same page” regarding data presented by the various Gear teams and with the BBF process as a whole.

### Process-led aspects of consensus-building

#### Reference to operation manual

When there were uncertainties in the discussion, the BBF director and committee members referenced the BBF operational manual. This manual detailed how the meetings should be conducted and offered examples of the scoring and recommendation process.

“*If I look at page 73 in the manual*, *it says master trainers have received National or International Certifications as Breastfeeding Specialist or Lactation Consultant*.”

### Process-led aspects of consensus-building

#### Asking questions

Throughout meetings, committee members often asked questions to the group. These questions were used to gauge the thoughts and opinions of the group and facilitate decision-making:

“*Are there any questions about this session*? *Any questions for that first gear*?”

In some cases, a question was asked to gain Samoa-specific information, data or knowledge from members’ expertise:

“*Can I ask if anyone can answer the question*? *If there is a trained or certified lactation consultant now in Samoa*, *or specialist*?”

In other cases, committee members asked questions to clarify a concept or improve their own understanding. This sometimes involved relying on others’ knowledge:

“*I think the word is formative research*. *Can you help [name of BBF expert]*? *Formative research is research that is evidence-based*?”

### Other consensus-building methods

The meeting transcripts revealed other consensus-building methods that were not included in the *a priori* framework [5]. These methods included a) using other countries who completed the BBF initiative as examples (“Using other countries as examples”); b) consulting with individuals who were not committee members, but who were present at meetings and had close involvement with the BBF process (“Receive “outside” help”); and c) invoking ideas of community and togetherness to engage committee members and keep them invested in the meeting process. The themes “Effective leader,” and “Other countries as examples” were “process-led”, while “Receive outside help” and “Community or togetherness” reflected methods that emerged “organically” during the meeting process.

### Process-led aspects of consensus-building

#### Other countries as examples

Committee members oftentimes brought up BBF initiatives in other countries to learn from or explain the BBF scoring process and program implementation. This strategy was used frequently, as in the follwing example during Meeting 1, when the committee chair used Ghana’s BBF experience as an example of how the Training and Program Delivery Gear was scored.

*Committee chair*: *“You should use examples because…., page 73, 74, in Ghana, there are multiple programs and institutions that conduct training, however there is no single institution to coordinate breastfeeding training, and the situation is fragmented.”*

### Organic aspects of consensus-building

#### Receive “outside” help

This subtheme reflects intervention from experts outside of the main committee, but who are familiar with the BBF process. For example, author (NH) from Yale University joined the discussion on scoring and presentation of data for a particular Gear during Meeting 2:

*Committee Member*: *[to NH] Do you have anything to say about our scoring here, or are there any gaps that you think that might help with our presentation?*

*NH*: *So I think the major questions* [to consider, moving forward] *are*: *if there any other data sources that should have been considered*, *whether the other people in the committee can think of more sources*, *and then*, *do we agree with the scoring or is there any debate [about the scoring]*?*”*

#### Community and togetherness

The committee leaders and members frequently invoked ideas of community, teamwork, and togetherness. They often described the committee itself as a “team” and that the BBF process and breastfeeding improvement in Samoa required “teamwork”, was “a collaborative effort” and “was a great goal” for all. During Meeting 1 on the Training and Program Delivery Gear, the committee leader described how coordinated efforts are essential to improving breastfeeding-related training on a national level in Samoa:

“*We are very thankful*, *and we are very happy that we have a group of people who are willing to push breastfeeding to the next level*. *And for Samoa*, *it’s a great goal for us to achieve with regards to our non-communicable disease crisis*. *Breastfeeding will help to reduce NCD [prevalence] among young children*.”

## Discussion

In this study, we explored the question: how did the 2018 BBF Samoa committee build consensus and make decisions? Following BBF processes in other countries [15, 16], the BBF Samoa initiative was particularly successful in that a) it led to the full support of six highly-prioritized recommendations for breastfeeding scale-up, b) it was followed by the signing of a breastfeeding policy that had been in development in Samoa for many years, and c) the BBF initiative and policy signing led to increased adherence to the Baby Friendly Hospital Initiative (BFHI) in Samoa’s two primary hospitals [7].

Through inductive and deductive coding, we identified 6 themes, 2 meta-subthemes, and 16 subthemes in total, all of which largely overlapped with the *a priori* operational framework describing successful consensus-building [5, 14]. Overall, the committee built consensus in one of two manners (the two meta-subthemes), 1) in ways prescribed by the BBF process itself or 2) in ways that were specific to this committee and emerged organically and independently of the prescribed BBF process.

First, we suggest that the structured or “process-led” meta-theme of consensus-building played a key role in facilitating the initiative’s success. Earlier work on decision-making theory in the 1970s and 80s described how providing groups with specific guidelines and adequately “structuring” a decision-making process can render the process simpler and more expedient. For instance, experiments have shown that when an “instructed” group (where a highly specific list of instructions were provided to aid decision making) and an “uninstructed” (control) groups were asked to complete a decision making task, the instructed group produced more high-quality decisions, exhibited greater creativity, and exerted greater caution when evaluating available evidence and making decisions [17–20]. In contrast, uninstructed groups tended to produce less high-quality decisions, have more inter-member conflicts, and exhibit a tendency to “rush” towards reaching a decision at all costs, often resorting to techniques such as majority rule [17–20].

Several themes in this study reflect the power of “instruction” as explored by earlier researchers [17, 19]. For instance, the adherence to an agenda at the start of each meeting and frequent reference to the BBF operational manual allowed the BBF Samoa committee to successfully make decisions about scoring and recommendations. Moreover, the effectiveness of the BBF committee chair (CSU) as a leader and her extensive knowledge of the relevant Samoan data as it applied to the BBF Gears ensured that the decision-making process went smoothly, without confusion or misunderstandings, and that time constraints were recognized and adhered to. The BBF initiative–with prescribed meeting structures, meeting-specific agendas and an operational manual–provides the necessary “instructions” or “blueprint” that allowed for successful consensus-building or decision-making during 2018 initiative.

Second, organic and committee-specific ways of building consensus contributed to the success of the BBF Samoa initiative. For example, committee members often used personal anecdotes to illustrate ideas and facilitate discussion, thus invoking their own experiences with breastfeeding and infant care as a method to guide decision-making. These findings reflect results of previous research, which has shown how personal stakeholder experiences shape their involvement in decision-making processes related to maternal and child health. In a large qualitative study of clinicians and staff across four health sectors in the U.S., authors found that stakeholders’ own experiences with breastfeeding, and the belief that breastfeeding and infant care was a personal/emotional issue, influenced their support for breastfeeding policy development [21]. Along similar lines, the use of humor and sarcasm throughout the meetings may have helped to keep committee members engaged and involved. Humor has been shown to be a tool for group problem-solving and leadership by relieving stress, reducing conflict, and facilitating new ideas [22]. In the BBF Samoa meetings, the sharing of personal anecdotes may have helped to engage committee members [5] by further accentuating the importance of breastfeeding scale-up and bringing the various topics and data presented during the meetings to life.

Values of community and togetherness may have also contributed to the decision-making process in BBF Samoa. Social values play a large role in shaping decision-making in health policy. For instance in Chile and Columbia, values of individuality, free choice and human dignity shaped policymakers’ decisions on legislation regulating private health insurance [23]. By framing the BBF committee as a community effort may have strengthened committee members’ engagement, thus aiding the consensus-building process.

There are several limitations of this study that warrant consideration. First, the audio files for Meeting 3 of the BBF Samoa process could not be located. During Meeting 3, the BBF committee presented the benchmark scores, reached final consensus on the benchmark scores, and calculated the gear scores. Thus, by not analyzing Meeting 3 audio transcripts, key parts of the consensus-building process may not have been captured by our qualitative analysis. We did, however, have access to detailed Meeting 3 meeting minutes, which allowed us to capture relevant outcomes from the meeting. Second, parts of the audio files had low quality, resulting in possible errors with transcription and translation. At times, this limited our ability to assess and understand the full conversation dynamics, including who was speaking. Third, these audio files only captured the main BBF meetings and not the gear team meetings or more informal conversations amongst committee members that happened outside of the designated meeting times. Despite these limitations, the loss of key information was minimized since BBF meetings are highly iterative and hence need to refer to each other throughout the process.

## Conclusion

This study examined the consensus-building process during the 2018 Becoming Breastfeeding Initiative (BBF) in Samoa. Utilizing an *a priori* framework for understanding successful consensus-building, we conducted an inductive and deductive qualitative content analysis of transcribed and translated audio recordings of the BBF meetings, during which a 20-member committee met to reach consensus on scores of gears and benchmarks for breastfeeding scale-up in Samoa. Sixteen themes emerged from the transcripts, including, but not limited to: the use of humor, intentional agendas, values of community and togetherness, effective leader, and reliance on expertise. These themes encapsulated the various parts of successful consensus-building outlined by the *a priori* framework. The themes also represented both purposive and organic ways in which consensus was built. The detailed manualization of the BBF process, its reliance on data, and its transparent and engaged committee were key for reaching consensus on BBF scores and recommendations in Samoa. These findings contribute to our understanding of decision-making within the BBF initiative and underscore the group strategies that may have resulted in government policy action, which can be applied in future policy scale-ups.
